# Advances in computer-assisted syndrome recognition by the example of inborn errors of metabolism

**DOI:** 10.1007/s10545-018-0174-3

**Published:** 2018-04-05

**Authors:** Jean T. Pantel, Max Zhao, Martin A. Mensah, Nurulhuda Hajjir, Tzung-Chien Hsieh, Yair Hanani, Nicole Fleischer, Tom Kamphans, Stefan Mundlos, Yaron Gurovich, Peter M. Krawitz

**Affiliations:** 1 Institute of Human Genetics and Medical Genetics, Charité - Universitätsmedizin Berlin, corporate member of Freie Universität Berlin, Humboldt-Universität zu Berlin, and Berlin Institute of Health, Berlin, Germany; 2 Institute for Genomic Statistics and Bioinformatics, University Hospital Bonn, Rheinische Friedrich-Wilhelms-Universität Bonn, Bonn, Germany; 3 Berlin Institute of Health (BIH), Anna-Louisa-Karsch 2, 10178 Berlin, Germany; 4 FDNA, Boston, MA USA; 5 GeneTalk, Bonn, Germany

## Abstract

Significant improvements in automated image analysis have been achieved in recent years and tools are now increasingly being used in computer-assisted syndromology. However, the ability to recognize a syndromic facial gestalt might depend on the syndrome and may also be confounded by severity of phenotype, size of available training sets, ethnicity, age, and sex. Therefore, benchmarking and comparing the performance of deep-learned classification processes is inherently difficult. For a systematic analysis of these influencing factors we chose the lysosomal storage diseases mucolipidosis as well as mucopolysaccharidosis type I and II that are known for their wide and overlapping phenotypic spectra. For a dysmorphic comparison we used Smith-Lemli-Opitz syndrome as another inborn error of metabolism and Nicolaides-Baraitser syndrome as another disorder that is also characterized by coarse facies. A classifier that was trained on these five cohorts, comprising 289 patients in total, achieved a mean accuracy of 62%. We also developed a simulation framework to analyze the effect of potential confounders, such as cohort size, age, sex, or ethnic background on the distinguishability of phenotypes. We found that the true positive rate increases for all analyzed disorders for growing cohorts (n = [10...40]) while ethnicity and sex have no significant influence. The dynamics of the accuracies strongly suggest that the maximum distinguishability is a phenotype-specific value, which has not been reached yet for any of the studied disorders. This should also be a motivation to further intensify data sharing efforts, as computer-assisted syndrome classification can still be improved by enlarging the available training sets.

**Electronic supplementary material:**

The online version of this article (10.1007/s10545-018-0174-3) contains supplementary material, which is available to authorized users.

## Introduction

In syndromology the information content of the facial gestalt is so extraordinarily high that photographs are important in the diagnostic work-up. This also holds true for many inborn errors of metabolism that result in dysmorphic facial features (see also a corresponding list from IEMbase© in the Supplemental material). Recently, advances in computer vision improved pattern recognition on ordinary facial photos of syndromic patients (Boehringer et al 2006; Ferry et al 2014; Gurovich et al 2018). These approaches also have the potential to quantify the similarities of patients to any specific syndrome for which a model exists and to decide whether there is a significant difference between gene-phenotypes (Knaus et al 2018; Gurovich et al 2018).

Face2Gene (FDNA Inc., Boston MA, USA) is such a novel tool that supports pattern recognition in frontal photographs (https://face2gene.com). The facial analysis within Face2Gene is a deep convolutional neural network (DCNN) that is referred to as DeepGestalt. Currently, this DCNN is able to compare a photo to about 300 different syndromic phenotype models and to compute a similarity value (Gurovich et al 2018). The CLINIC application of Face2Gene provides a list of 30 differential diagnoses that are based on these gestalt scores.

While Face2Gene CLINIC makes the latest classification models available that were trained on the entire set of suitable cases the user community provided, a recently launched application, referred by RESEARCH, allows working with DeepGestalt in a controllable environment (Knaus et al 2018). This app can be used to learn the facial gestalts of different cohorts that share for example disease-causing mutations in the same gene or pathway. The results of an experiment are gestalt models suitable for binary and multi-class comparisons. The true positive rates (TPRs) as well as the error rates of the multi-class problem are reported in a confusion matrix, whereas the pairwise comparison of cohorts are evaluated as receiver operating characteristics (ROC) curves.

If the gestalt models achieve accuracies in the classification of photographs higher than randomly expected, there are recognizable facial patterns in individuals of a cohort. When phenotypes of the same molecular subgroup are compared, a significant distinguishability also means that a clinical entity can be delineated based on the facial gestalt. While this delineation of syndromic phenotypes has been reserved to a few experts in the field, computer-assisted pattern recognition might help to objectify, even quantify this process. However, if we interpret the accuracy of a classifier as the quantification of the distinguishability of disease-phenotypes, it is of utmost importance that the factor we are measuring is not confounded by, e.g., age, ethnicity or sex. In this work, we therefore present a framework for a systematic analysis of potential confounders that we tested on patients with inborn errors of metabolism (IEMs).

## Patients and methods

We focused our analysis on IEMs and phenotypically similar disorders, 1) that have a high prevalence, 2) that are already represented in Face2Gene CLINIC, and 3) that are straightforward to confirm in the lab (Baehner et al 2005). We compiled an original sample set of 289 typical and atypical patients with mucoploysaccharidosis (MPS I and II), mucolipidosis (ML II alpha/beta and ML III alpha/beta), Smith-Lemli-Opitz syndrome (SLOS), and Nicolaides-Baraitser syndrome (NCBRS) that have all been molecularly confirmed (see Supplemental material for literature references). The facial gestalts of some patients are so similar, even for experts, that it is hard to tell the diseases apart without enzymatic or genetic testing. Due to this phenotypic overlap, our data set is also a challenging task for computer vision. In addition, especially within the IEMs, there is considerable phenotypic variability. For the lysosomal storage disorders (LSD) MPS and ML, hardly any symptoms are present at birth, but they usually appear during early childhood and progress during adolescence (Muenzer 2011). The extent of the enzyme deficiency influences the severity of the phenotype and in, e.g., MPS I, the genotype-phenotype correlations are also reflected by the clinical subdivision into Hurler, Hurler-Scheie, and Scheie syndrome (Bunge et al 1998). Although there is no cure for MPS, hematopoietic stem cell transplants or enzyme replacement therapies (ERT) have shown considerable treatment success that could also slow down the progression of symptoms (Kung et al 2013, 2015; Watson et al 2014; Bradley et al 2017; Kubaski et al 2017; Rodgers et al 2017). This also means treatment duration in addition to age might affect the severity of the phenotype in this disease cohort.

**Fig. 1:**
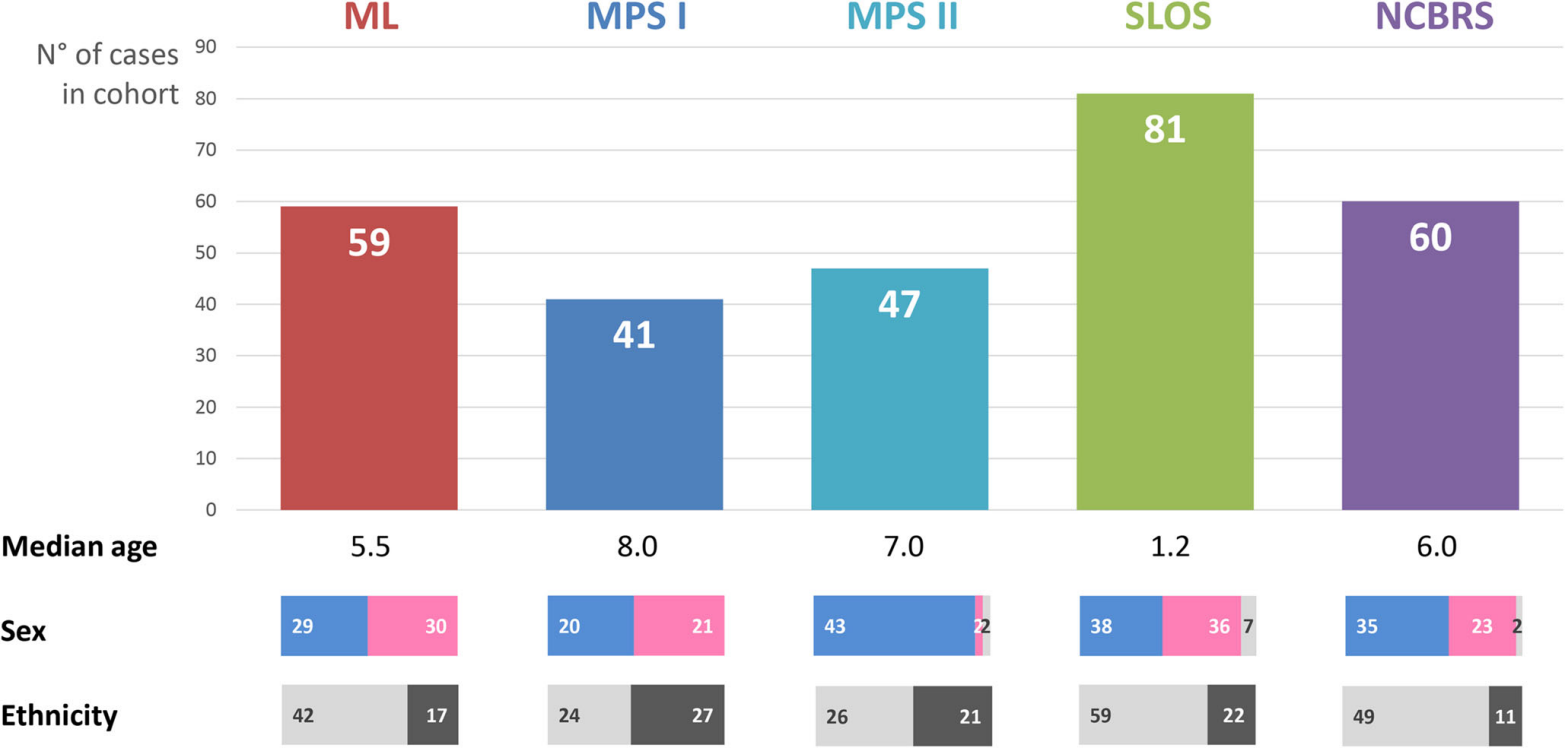
Overview of the original sample set with sex ratios (male/female/sex not mentioned) and ethnic backgrounds of European (left) vs. Non-European (right)

For a systematic analysis of confounders, we annotated for each photo the corresponding age, sex, ethnic background, and treatment status of the patient. If available, the disease-causing mutations were recorded in HGVS nomenclature and the phenotypic features were annotated in HPO terminology. A summary of the analyzed samples is shown in Fig. 1. The entire case-based data collection is part of a larger knowledge base, called deep phenotyping for deep learning, DPDL, that can be accessed upon request and that serves as a set for computer-assisted image analysis benchmarking.

The phenotypic comparison of the cohorts was based on the clinical or molecular diagnosis and all experiments were run in Face2Gene’s RESEARCH application (version 17.6.2), which is accessible to registered users.

With our original sample set of 289 labeled photos, we were able to study the potential confounders cohort size, ethnic background, and sex. For an analysis of the intertwined factors age and treatment duration, we did not have sufficient individuals to form the required subsets. The performance on subsets was evaluated after random down-sampling to the same size. Based on the python requests library v2.18.4, we built a framework to automatize the repetition of experiments and the TPRs of the resulting confusion matrices were averaged over five iterations for each setting. The scripts for the simulations are available on request and can be used to reproduce the results.

The influence of the cohort size was analyzed by incrementing evenly sized subsets from 10 to 40. The change of the performance was fitted to a linear model and analyzed for significance using *linregress* of the SciPy library. The other potential cofounders, ethnic background and sex, were analyzed by excluding cohort size as a covariate. For these experiments, we sampled each cohort down to the greatest common size for each potential confounder. The greatest common size for the potential confounder male sex would, e.g., be 20, because there are only 20 male patients with MPS I in our original sample set (see Fig. 1). By this means cohort size has no influence on the performance and allowed an analysis of the potential confounders sex and ethnicity. Matthews correlation coefficient (MCC) is a measure of the quality of a two-class classification. Therefore, we reduced the multiclass confusion matrix to a two-class matrix for every diagnosis. Then we calculated the mean MCC for all iterations of the same experiment. If the difference of the MCCs of the potential confounder and control experiments was within the range of two standard deviations of the MCCs of the control experiments, we regarded the variable as not having a significant effect on the analyzed disease.

## Results

### Classification of the original sample set in Face2Gene CLINIC and RESEARCH

**Fig. 2:**
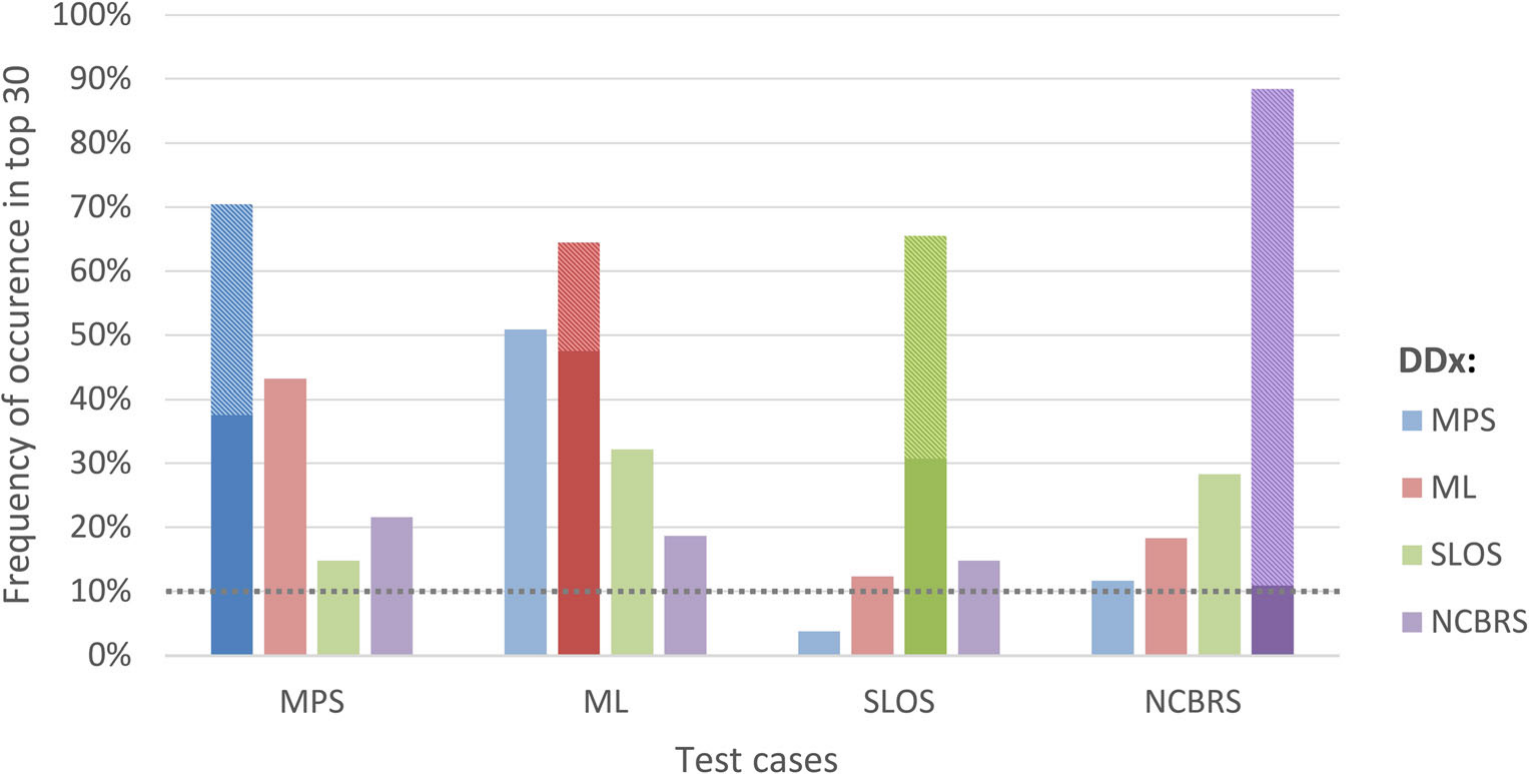
Frequency of occurrence of the five disorders as differential diagnoses (DDx) under the first 30 ranks in Face2Gene CLINIC in the respective test groups. The proportion of the correct diagnosis at the first rank is hatched. For instance, the correct diagnosis “MPS” appears in the MPS I and II cohort in 34% of the cases at the top position and in altogether 70% in the top 30. With about 300 DDx to choose from in gestalt match a frequency of occurrence above 10% in the top 30 ranks (dotted line) indicates phenotypic similarity

Face2Gene CLINIC lists the 30 most likely differential diagnoses (DDx) per case. If only a frontal facial photograph is uploaded, and no clinical features are annotated, these DDx represent syndromes that achieved the highest gestalt scores in the image analysis. Figure 2 shows the frequency of MPS, ML, SLOS, and NCBRS in the respective test cohort among these 30 suggested diagnoses. MPS I and MPS II were combined in Face2Gene CLINIC under the phenotypic series of MPS. The correct diagnosis was reported among these top 30 DDx in more than 60% of the cases for all five test cohorts.

**Fig. 3:**
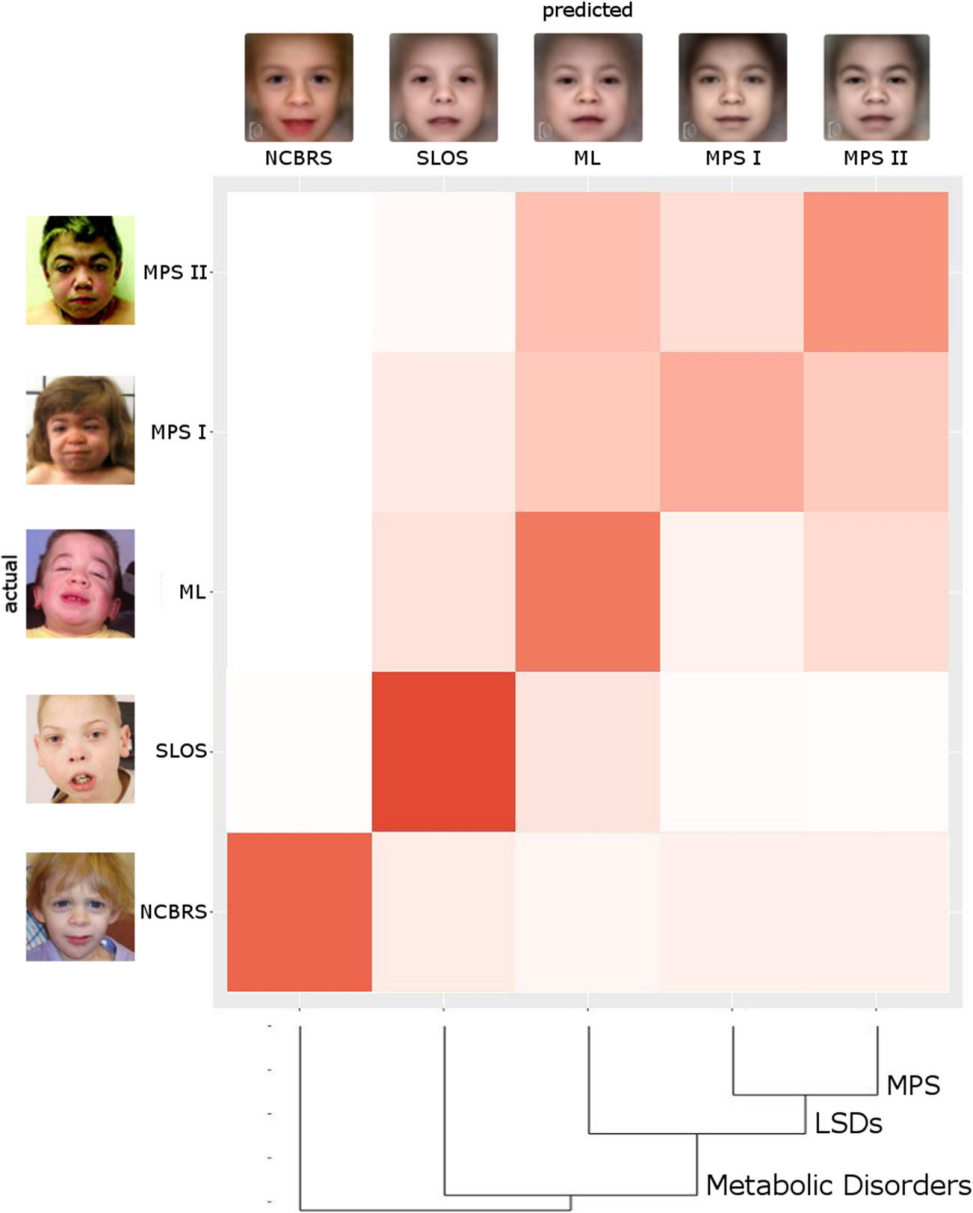
The performance of the gestalt-model in the multi-class problem in Face2Gene RESEARCH is shown as a color-coded confusion matrix, where deep red corresponds to a high value. True positive rates (TPR) are on the diagonal and false negatives and positives rates aside. The whole classification process achieves an accuracy of 62%, which is significantly better than randomly expected (28%). Syndrome masks on top show the average appearance of the disorder, while photos on the left show instances of individuals featuring the respective disorder. The dendrogram is the result of a clustering analysis and visualizes the similarity of the disorders

With about 300 phenotypic models to choose from, a frequency of occurrence above 10% in the top 30 for a DD that is not the correct diagnosis can also be interpreted as similarity. Not surprisingly, MPS and ML appear in more than 40% of the cases as mutual DDx, mirroring their high phenotypic similarity. While the patient data that was used for modeling the different phenotypes in Face2Gene CLINIC is not directly accessible, Face2Gene RESEARCH allows in silico experiments with user defined cohorts. The resulting confusion matrix for the original sample set is shown in Fig. 3 as a heat map. The stronger the field is colored, the higher the probability for an actual test case to end up in the respective predicted class. The high values on the diagonal for all cohorts illustrate the power of DeepGestalt to distinguish even similar phenotypes. Based on the probability values of the confusion matrix, we calculated a dendrogram. The three lysosomal storage disorders MPS I, MPS II, and ML are close, while the distance to NCBRS that is also often described by coarse facial features is largest.

### Influence of growing cohort size on classification accuracy

**Fig. 4:**
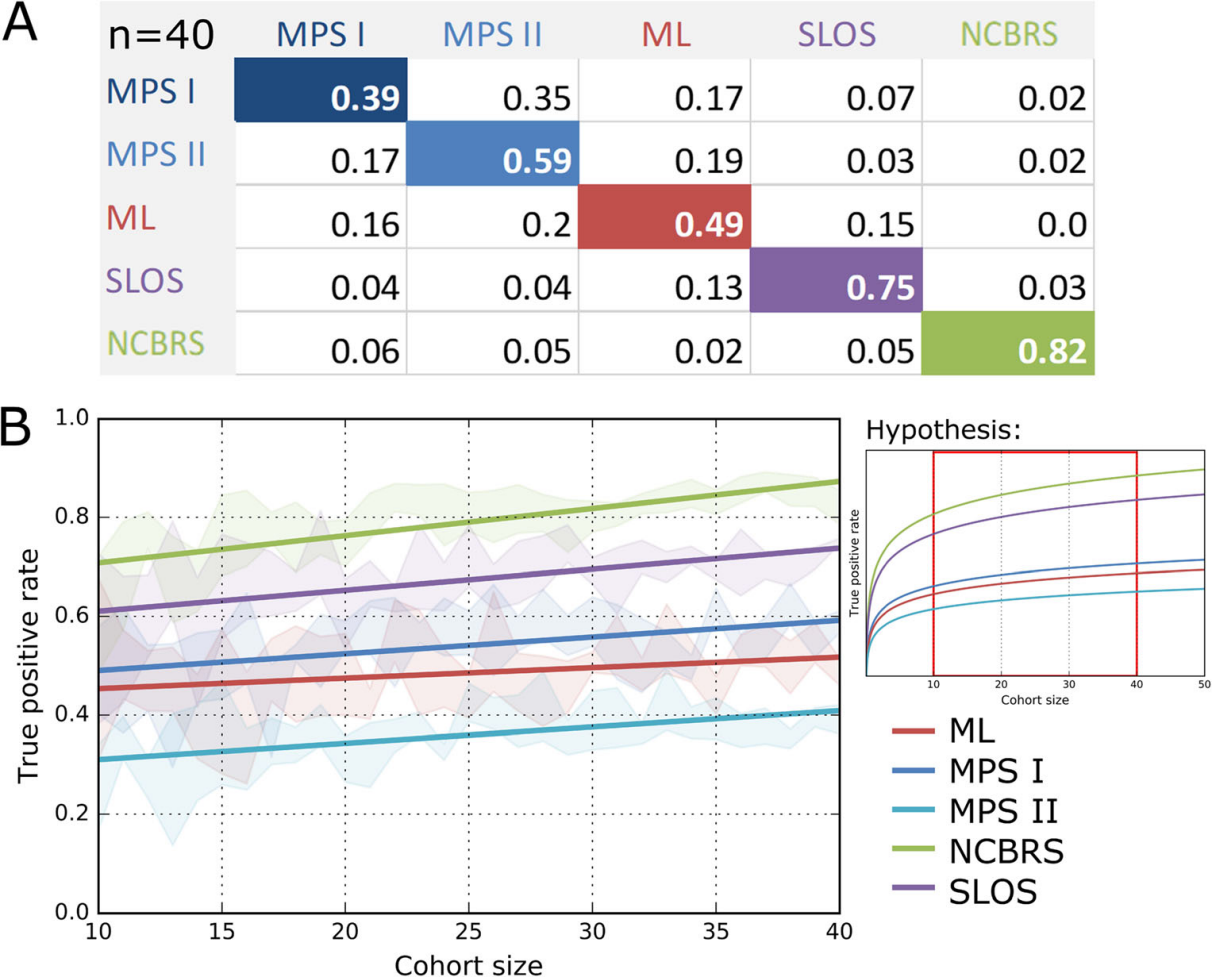
(**a**) Confusion matrix with TPRs and FPRs with a cohort size of *n* = 40. (**b**) Course of TPRs with increasing cohort size with linear regression. The performance of the classification process was evaluated for equally sized cohorts from *n* = 10 to n = 40. The true positive rates for the prediction of the disorder improve with increasing cohort size and seem to approach different limits, indicating a difference in relative distinguishability. Especially the prediction of SLOS and NCBRS benefit, when the classifier is trained on more cases. The inference of the correct lipid storage disorder increases less for larger cohort sizes

To analyze the influence of cohort size on the performance of the classification process, we increased the number of individuals per group stepwise from 10 to 40. TPRs with cohorts of only ten individuals were already higher than randomly expected and increased for all phenotypes with a growing cohort size, while the standard deviation decreased (Fig. 4). The dynamics of the TPRs were best fitted with a linear function and indicate that the full potential of computer-assisted classification has not been reached yet with the available image data. However, we hypothesize that the number of images needed to reach a maximum in the distinguishability could be different for each syndrome and might depend on the clinical variability of the phenotype. The TPRs of NCBRS and SLOS are the highest in comparison with the other cohorts. The inborn errors of metabolism are more frequently misclassified among each other than as SLOS or NCBRS. Notably, the MPS I-TPR nearly equals the fraction of MPS I cases falsely classified as MPS II. It is noteworthy that ML is falsely classified as SLOS in around 14% and vice versa.

### Effect of ethnic background or sex on performance

We hypothesized, that a bias in the setup of the cohorts with respect to the ethnic background or the sex, might affect the performance. In general, the performance should drop if a true confounder is removed. If the performance increases instead after splitting up cohorts, this in an indicator that there is some characteristic feature that can be more efficiently learned in a more homogeneous group of patients.

Lumaka et al discussed in their study that certain features of Down syndrome, such as a deep nasal bridge and thick upper lips, are less prominent in individuals of African descent (Lumaka et al 2017). We adjusted for the same cohort size (*n* = 19) and computed the MCCs that could be achieved in the classification of DS patients from Sub-Saharan Africa or Central Europe (Table 1). Interestingly, we observed a substantially better performance for the DS model that was trained on the same ethnic background compared to a mixed setup of the cohorts (ΔMCC/STD for AFR vs. Mixed: 3.75 and ΔMCC/STD for CEU vs. Mixed: 2.70). This finding supports the hypothesis of a slightly different facial appearance of DS in Europeans and Africans.

**Table 1:** The classification of DS is more accurate on only European or African patients. These marked differences cannot be observed for ML, MPS I, MPS II, SLOS, and NCBRS. Also, the restriction to only male patients has only a minor effect on the performance. The difference of MCCs for the binary classification of every disease was normalized by the standard deviations of MCCs that were computed in the mixed controls

	pt. confounder	DS	pt. confounder	ML	MPS I	MPS II	SLOS	NCBRS
	CEU vs mixed:	2.7	CEU vs mixed:	−0.7	1.53	−0.29	0.31	0.77
ΔΜCC-STD	AFR vs mixed:	3.75	male vs mixed:	0.14	0.13	1.17	1.84	1.12

In contrast to DS, we did not observe such marked differences in the MCCs for MPS I, MPS II, ML, SLOS, and NCBRS, when running the experiments for *n* = 22 cohorts that consisted only of European patients. An analysis for another background in these disorders was not possible due to a lack of sufficient patients.

Another potential confounder in the five-class problem of MPS I, MPS II, ML, SLOS, and NCBRS that we analyzed is sex. All but two of the MPS II patients were male, whereas the sex ratios for the other disorders were close to 1. This means knowing the sex would help with distinguishing MPS II from MPS I cases. Interestingly, however, the MCC for the MPS II classification did not decrease, when all other cohorts were also restricted to male patients only and same cohort sizes of *n* = 20. This indicates that a bias in the sex ratios does not affect the performance of the classification process substantially for the tested syndromes.

## Discussion

### General distinguishability

The TPRs that were achieved for all disorders in the five-class problems were higher than expected by random chance. Thus, our results show that the FDNA technology is capable of delineating gestalt differences even for clinically similar phenotypes. This finding is especially remarkable for the phenotypes of MPS and ML and is also supported by high AUROC values in binary classifications (Suppl. Fig. 1).

The difference in TPRs for the syndromes could be interpreted as different recognizabilities. Notably, SLOS and NCBRS are more recognizable than MPS I, MPS II, and ML. This corresponds to the results from the CLINIC app, where ML and MPS show lower distinguishability. These findings are in agreement with geneticist expert opinion, who label ML as highly similar to MPS.

The high TPRs found in our analyses corresponds to the results of two other studies on phenotypes of molecular pathway disorders. For Noonan syndrome as well as for GPI-anchor deficiencies, significant phenotypic substructures could be detected. This also illustrates that an even more fine-grained phenotype modeling might be possible with the CLINIC app in the future.

### Ethnicity

Distinguishing MPS I from the other disorders was slightly more effective when working in a European background. A possible explanation for this slight increase in performance might be that there are certain features that are restricted or more prominent in European patients and that might therefore be learned more effectively if relatively more cases are used for training the model. This issue has already been discussed for other disorders, such as Fragile-X syndrome and Down syndrome, were ethnic specific differences in the feature presentation are known (Schwartz et al 1988; Lumaka et al 2017). Although we could replicate these effects for DS, we did not see a prominent change in the performance in the other phenotypes, which indicates that that ethnic background is not a strong confounder in the classification process.

### Sex

The human face shows a sexual dimorphism, possibly even at an early age, making sex a potential confounder in any facial image analysis process (Zhang et al 2016). The classification accuracies in our experiments that were based on data sets adjusted to individuals of the same sex, did not significantly differ, suggesting that the classification method is robust to sex as a confounder. Also, the mean MCCs showed no significant change when training the classifier on only male individuals as compared to a training cohort consisting of both sexes. Our interpretation is that sex does not confound the classification of MPS I, MPS II, ML, SLOS, and NCBRS.

### Benchmarking

We are just beginning to understand the potential of computer-assisted image analysis in the field of syndromology. In this work we have presented a general approach to study the distinguishability of a phenotype and to test the confounding effect of variables such as ethnicity or sex. We have applied this framework to a selection of inborn errors of metabolism, however, in principle, it is applicable to any other disorders.

It would also be interesting to compare the performance of the FDNA technology to the accuracies of other, previously published approaches of automated image analysis of syndromic patients. Comparative evaluation, however, is impeded by the lack of a publicly available data set for benchmarking. Earlier benchmarking approaches merely relied on the comparison to a human classification performance. To achieve an objective evaluation of computer vision, we strongly advocate to build a resource for image data of molecularly confirmed syndromic cases.

### Conclusion and outlook

In this work we report on a next-generation phenotyping technology that can be used to study the similarities and differences between patients with rare genetic disorders. The framework that we present is not only suited to measure the accuracies of the DCNN in the classification process but also to test for confounding effects. Especially with respect to the novel and powerful methods in artificial intelligence, it is crucial to learn more about what is actually quantified by a DCNN. Our results show that DeepGestalt, the next-generation-phenotyping technology within Face2Gene, is not confounded by sex or ethnic background for the studied phenotypes. The high predictive value for IEMs in the CLINIC application also makes Face2Gene a valuable tool to detect these kinds of disorders. This is especially of importance for patients that might have evaded an early detection by new born screenings. The importance of such programs is, however, untouched as the outcome improves the earlier ERT can be started and the evolving phenotype of IEMs might be more difficult to detect in newborns than in older age groups.

Apart from detection, an even more important role of computer vision could be disease monitoring if a neural network is not only able to sense the presence of a disease but also to quantify features that, e.g., mirror the progress of GAG deposition. We hope to be able to investigate this question in future research when more data becomes available.

## Electronic supplementary material

ESM 1 (DOCX 27 kb)

ESM 2 (PNG 170 kb)

## Abbreviations

AFR: African
CEU: Central European
DDx: Differential diagnoses
DPDL: Deep phenotyping for deep learning
DS: Down syndrome
ERT: Enzyme replacement therapy
FDNA: Facial dysmorphology novel analysis
FNR: False negative rate
FPR: False positive rate
GAG: Glycosaminoglycan
HPO: Human phenotype ontology
LSD: Lysosomal storage disease
ML: Mucolipidosis
MPS I: Mucopolysaccharidosis type I
MPS II: Mucopolysaccharidosis type II
NCBRS: Nicolaides-Baraitser syndrome
ROC: Receiver operating characteristics
SLOS: Smith-Lemli-Opitz syndrome
TPR: True positive rate

## References

[CR1] Baehner F, Schmiedeskamp C, Krummenauer F (2005). Cumulative incidence rates of the mucopolysaccharidoses in Germany. J Inherit Metab Dis.

[CR2] Boehringer S, Vollmar T, Tasse C (2006). Syndrome identification based on 2D analysis software. Eur J Hum Genet.

[CR3] Bradley LA, Haddow HRM, Palomaki GE (2017) Treatment of mucopolysaccharidosis type II (hunter syndrome): results from a systematic evidence review. Genet Med. 10.1038/gim.2017.30 10.1038/gim.2017.30 28640238

[CR4] Bunge S, Clements PR, Byers S (1998). Genotype-phenotype correlations in mucopolysaccharidosis type I using enzyme kinetics, immunoquantification and in vitro turnover studies. Biochim Biophys Acta.

[CR5] Ferry Q, Steinberg J, Webber C et al (2014) Diagnostically relevant facial gestalt information from ordinary photos. Elife 3:e02020 10.7554/eLife.02020 PMC4067075 24963138

[CR6] Gurovich Y, Hanani Y, Bar O et al (2018) DeepGestalt — identifying rare genetic syndromes using deep learning. arXiv:1801.07637

[CR7] Knaus A, Pantel JT, Pendziwiat M (2018). Characterization of glycosylphosphatidylinositol biosynthesis defects by clinical features, flow cytometry, and automated image analysis. Genome Med.

[CR8] Kubaski F, Yabe H, Suzuki Y et al (2017) Hematopoietic stem cell transplantation for patients with mucopolysaccharidosis II. Biol Blood Marrow Transpl. 10.1016/j.bbmt.2017.06.020 10.1016/j.bbmt.2017.06.020 PMC5659208 28673849

[CR9] Kung S, Walters M, Claes P et al (2013) A dysmorphometric analysis to investigate facial phenotypic signatures as a foundation for non-invasive monitoring of lysosomal storage disorders. JIMD Rep 8:31–39. 10.1007/8904_2012_152 10.1007/8904_2012_152 PMC3565667 23430517

[CR10] Kung S, Walters M, Claes P et al (2015) Monitoring of therapy for mucopolysaccharidosis type I using dysmorphometric facial phenotypic signatures. JIMD Rep 22:99–106. 10.1007/8904_2015_417 10.1007/8904_2015_417 PMC4486281 25732999

[CR11] Lumaka A, Cosemans N, Lulebo Mampasi A (2017). Facial dysmorphism is influenced by ethnic background of the patient and of the evaluator. Clin Genet.

[CR12] Muenzer J (2011). Overview of the mucopolysaccharidoses. Rheumatol.

[CR13] Rodgers NJ, Kaizer AM, Miller WP (2017). Mortality after hematopoietic stem cell transplantation for severe mucopolysaccharidosis type I: the 30-year University of Minnesota experience. J Inherit Metab Dis.

[CR14] Schwartz CE, Phelan MC, Pulliam LH (1988). Fragile X syndrome: incidence, clinical and cytogenetic findings in the black and white populations of South Carolina. Am J Med Genet.

[CR15] Watson HA, Holley RJ, Langford-Smith KJ (2014). Heparan sulfate inhibits hematopoietic stem and progenitor cell migration and engraftment in mucopolysaccharidosis I. J Biol Chem.

[CR16] Zhang W, Smith ML, Smith LN, Farooq A (2016). Gender recognition from facial images: two or three dimensions?. J Opt Soc Am A Opt Image Sci Vis.

