# Supplementary material for: Advances in computer-assisted syndrome recognition by the example of inborn errors of metabolism
Source: J Inherit Metab Dis. 2018 Apr 5;41(3):533–9. doi: 10.1007/s10545-018-0174-3 (PMC5959962; doi:10.1007/s10545-018-0174-3)
Supplement: Supplementary file 1 — (DOCX 27 kb) [file 10545_2018_174_MOESM1_ESM.docx]

Supplemental Material

# Advances in computer-assisted syndrome recognition by the example of inborn errors of metabolism

Table 1: Genes and inherited errors of metabolism that are characterized by coarse facial features or facial dysmorphism in IEMbase© ([www.iembase.org](http://www.iembase.org)):

**Gene IEM**

FH Fumarase deficiency

DHCR24 Desmosterolosis

SGSH Sanfilippo A disease

NAGLU Sanfilippo B disease

HGSNAT Sanfilippo C disease

GNS Sanfilippo D disease

GALNS Morquio A diease

GLB1 Morquio B disease

ARSB Maroteaux-Laby disease

GUSB Sly disease

AGA Aspartylglucosaminuria

FUCA1 Fucosidosis

MAN2B1 Mannosidosis

MANBA Beta-mannosidosis

NAGA Kanzaki disease

NEU1 Sialidosis

SLC17A5 Salla disease

GLB1 GM1-gangliosidosis

GNPTAB Mucolipidosis II alpha/beta

GNPTG Mucolipidosis III gamma

SUMF1 Multiple sulfatase deficiency

CTSA Galactosialidosis

ALG3 Mannosyltransferase 6 deficiency

ALG12 Mannosyltransferase 8 deficiency

ALG8 Glucosyltransferase 2 deficiency

ALG2 Mannosyltransferase 2 deficiency

ALG1 Mannosyltransferase 1 deficiency

ALG9 Mannosyltransferase 7-9 deficiency

MGAT2 N-acetylglucosaminyltransferase 2 deficiency

GCS1 Glucosidase 1 deficiency

SRD5A Steroid 5 alpha-reductase 3 deficiency

B4GALT1 Beta-1,4-galactosyltransferase 1 deficiency

SLC35C1 GDP-ficpse transporter deficiency

DK1 Dolichol kinase deficiency DK1

COG7 Component of COG complex 7 deficiency

COG1 Component of COG complex 1 deficiency

B4GALT7 Beta-1,4-galactosyltransferase 7 deficiency

B3GALTL O-Fucose-specific beta-1,3-N-glucosyltransferase

ALG11 Mannosyltransferase 4-5 deficiency

COG6 Component of COG complex 6 deficiency

LRP130 Leigh Snydrome with French-Canadian Ethnicity

MRPS16 Combined Oxidative Phosphaorylation Defect 2

TMEM70 ATP synthase deficiency

GLUT10 Arterial tortuosity syndrome

LIPA Mucolipidosis III alpha/beta

NANS N-acetylneuraminic acid synthase deficiency

PGM3 Immunodeficiency-23

SSR4 Congenital disorder of glycosylation SSR4

COG2 Component of COG complex 2 deficiency

ATP6V1A Cutis laxa

ATP6V1E1 Cutis laxa

VPS33A Mucopolysaccharidosis-plus

UQCRC2 Mitochondrial complex III deficiency, nuclear type 5

**Patient photos were derived from the following publications:**

Abuelo DN, Tint GS, Kelley R, Batta AK, Shefer S, Salen G (1995) Prenatal detection of the cholesterol biosynthetic defect in the Smith-Lemli-Opitz syndrome by the analysis of amniotic fluid sterols. Am J Med Genet 56: 281-285.

Alfadhel M, AlShehhi W, Alshaalan H, Al Balwi M, Eyaid W (2013) Mucolipidosis II: first report from Saudi Arabia. Ann Saudi Med 33: 382-386.

Al-Owain M, Imtiaz F, Shuaib T, et al (2012) Smith-Lemli-Opitz syndrome among Arabs. Clin Genet 82: 165-172.

Amorin M, Carlin A, Protzel A (2012) [Mucopolysaccharidosis I, Hurler syndrome: a case report]. Arch Argent Pediatr 110: e103-106.

Anderson AJ, Stephan MJ, Walker WO, Kelley RI (1998) Variant RSH/Smith-Lemli-Opitz syndrome with atypical sterol metabolism. Am J Med Genet 78: 413-418.

Aynaci FM, Cakir E, Aynaci O (2002) A case of I-cell disease (mucolipidosis II) presenting with craniosynostosis. Childs Nerv Syst 18: 707-711.

Babovic-Vuksanovic D, Jacobson RM, Lindor NM, Weiler CR (2005) Selective antibody immune deficiency in a patient with Smith-Lemli-Opitz syndrome. J Inherit Metab Dis 28: 181-186.

Beck M (1983) Papilloedema in association with Hunter's syndrome. Br J Ophthalmol 67: 174-177.

Beck M, Barone R, Hoffmann R, et al (1995) Inter- and intrafamilial variability in mucolipidosis II (I-cell disease). Clin Genet 47: 191-199.

Beck M, Steglich C, Zabel B, et al (1992) Deletion of the Hunter gene and both DXS466 and DXS304 in a patient with mucopolysaccharidosis type II. Am J Med Genet 44: 100-103.

Berry R, Wilson H, Robinson J, et al (1989) Apparent Smith-Lemli-Opitz syndrome and Miller-Dieker syndrome in a family with segregating translocation t(7;17)(q34;p13.1). Am J Med Genet 34: 358-365.

Bijarnia S, Shaw P, Vimpani A, et al (2009) Combined enzyme replacement and haematopoietic stem cell transplantation in Hurler syndrome. J Paediatr Child Health 45: 469-472.

Bradbury JA, Martin L, Strachan IM (1989) Acquired Brown's syndrome associated with Hurler-Scheie's syndrome. Br J Ophthalmol 73: 305-308.

Brama I, Gay I, Feinmesser R, Springer C (1986) Upper airway obstruction in Hunter syndrome. Int J Pediatr Otorhinolaryngol 11: 229-235.

Bramswig NC, Ludecke HJ, Alanay Y, et al (2015) Exome sequencing unravels unexpected differential diagnoses in individuals with the tentative diagnosis of Coffin-Siris and Nicolaides-Baraitser syndromes. Hum Genet 134: 553-568.

Bukelis I, Porter FD, Zimmerman AW, Tierney E (2007) Smith-Lemli-Opitz syndrome and autism spectrum disorder. Am J Psychiatry 164: 1655-1661.

Burruss DM, Wood TC, Espinoza L, Dwivedi A, Holden KR (2012) Severe Hunter syndrome (mucopolysaccharidosis II) phenotype secondary to large deletion in the X chromosome encompassing IDS, FMR1, and AFF2 (FMR2). J Child Neurol 27: 786-790.

Burton BK, Giugliani R (2012) Diagnosing Hunter syndrome in pediatric practice: practical considerations and common pitfalls. Eur J Pediatr 171: 631-639.

Cardoso ML, Barbosa M, Serra D, et al (2014) Living with inborn errors of cholesterol biosynthesis: lessons from adult patients. Clin Genet 85: 184-188.

Castori M, Covaciu C, Rinaldi R, Grammatico P, Paradisi M (2008) A rare cause of syndromic hypotrichosis: Nicolaides-Baraitser syndrome. J Am Acad Dermatol 59: S92-98.

Cathey SS, Leroy JG, Wood T, et al (2010) Phenotype and genotype in mucolipidoses II and III alpha/beta: a study of 61 probands. J Med Genet 47: 38-48.

Chakraborty PP, Biswas SN, Ray S, Dey SK (2016) Mucopolysaccharidosis type I disguised as rickets. BMJ Case Rep 2016.

Chasalow FI, Blethen SL, Taysi K (1985) Possible abnormalities of steroid secretion in children with Smith-Lemli-Opitz syndrome and their parents. Steroids 46: 827-843.

Choi PT, Nowaczyk MJ (2000) Anesthetic considerations in Smith-Lemli-Opitz syndrome. Can J Anaesth 47: 556-561.

Colavita N, Orazi C, Fileni A, Leone PC, Ricci R, Segni G (1986) A further contribution to the knowledge of mucopolysaccharidosis I H/S compound. Presentation of two cases and review of the literature. Australas Radiol 30: 142-149.

DeBarber AE, Eroglu Y, Merkens LS, Pappu AS, Steiner RD (2011) Smith-Lemli-Opitz syndrome. Expert Rev Mol Med 13: e24.

Demirdoven M, Yazgan H, Korkmaz M, Gebesce A, Tonbul A (2014) Smith-lemli-opitz syndrome: a case with annular pancreas. Case Rep Pediatr 2014: 623926.

Digilio MC, Marino B, Giannotti A, Dallapiccola B, Opitz JM (2003) Specific congenital heart defects in RSH/Smith-Lemli-Opitz syndrome: postulated involvement of the sonic hedgehog pathway in syndromes with postaxial polydactyly or heterotaxia. Birth Defects Res A Clin Mol Teratol 67: 149-153.

Ejaz R, Babul-Hirji R, Chitayat D (2016) The evolving features of Nicolaides-Baraitser syndrome - a clinical report of a 20-year follow-up. Clin Case Rep 4: 351-355.

Engstler M, Reuter G, Schauer R (1993) The developmentally regulated trans-sialidase from Trypanosoma brucei sialylates the procyclic acidic repetitive protein. Mol Biochem Parasitol 61: 1-13.

Fahnehjelm KT, Tornquist AL, Malm G, Winiarski J (2006) Ocular findings in four children with mucopolysaccharidosis I-Hurler (MPS I-H) treated early with haematopoietic stem cell transplantation. Acta Ophthalmol Scand 84: 781-785.

Freisinger P, Padovani JC, Maroteaux P (1992) An atypical form of mucolipidosis III. J Med Genet 29: 834-836.

Gabrielli O, Clarke LA, Bruni S, Coppa GV (2010) Enzyme-replacement therapy in a 5-month-old boy with attenuated presymptomatic MPS I: 5-year follow-up. Pediatrics 125: e183-187.

Gana S, Panizzon M, Fongaro D, et al (2011) Nicolaides-Baraitser syndrome: two new cases with autism spectrum disorder. Clin Dysmorphol 20: 38-41.

Gericke GS (1977) Mucolipidosis III: two patients displaying genetic pleiotropism. S Afr Med J 51: 140-144.

Giugliani R, Villarreal ML, Valdez CA, et al (2014) Guidelines for diagnosis and treatment of Hunter Syndrome for clinicians in Latin America. Genet Mol Biol 37: 315-329.

Gordon N (1973) I-cell disease--mucolipidosis II. Postgrad Med J 49: 359-361.

Grant BP, Beard JS, de Castro F, Guiglia MC, Hall BD (1998) Extensive mongolian spots in an infant with Hurler syndrome. Arch Dermatol 134: 108-109.

Gripp KW, Baker L, Telegrafi A, Monaghan KG (2016) The role of objective facial analysis using FDNA in making diagnoses following whole exome analysis. Report of two patients with mutations in the BAF complex genes. Am J Med Genet A 170: 1754-1762.

Guio JA, Ramirez PA, Bermudez M, Obando FS (2012) [Mucolipidosis type II-inclusion cell disease]. An Pediatr (Barc) 76: 108-109.

Gupta A, Uttarilli A, Dalal A, Girisha KM (2015) Hunter syndrome with late age of presentation: clinical description of a case and review of the literature. BMJ Case Rep 2015.

Guven G, Cehreli ZC, Altun C, et al (2008) Mucopolysaccharidosis type I (Hurler syndrome): oral and radiographic findings and ultrastructural/chemical features of enamel and dentin. Oral Surg Oral Med Oral Pathol Oral Radiol Endod 105: 72-78.

Haas D, Armbrust S, Haas JP, et al (2005) Smith-Lemli-Opitz syndrome with a classical phenotype, oesophageal achalasia and borderline plasma sterol concentrations. J Inherit Metab Dis 28: 1191-1196.

Herd JK, Dvorak AD, Wiltse HE, Eisen JD, Kress BC, Miller AL (1978) Mucolipidosis type III. Multiple elevated serum and urine enzyme activities. Am J Dis Child 132: 1181-1186.

Holt JB, Poe MD, Escolar ML (2011) Natural progression of neurological disease in mucopolysaccharidosis type II. Pediatrics 127: e1258-1265.

Horiuchi R, Ishikawa H, Ishii Y, Watanabe Y, Noguchi T, Suzuki S (1976) Mucopolysaccharidosis with special reference to Scheie syndrome. J Dermatol 3: 171-178.

Huang Y, Bron AJ, Meek KM, Vellodi A, McDonald B (1996) Ultrastructural study of the cornea in a bone marrow-transplanted Hurler syndrome patient. Exp Eye Res 62: 377-387.

Irons M, Elias ER, Tint GS, et al (1994) Abnormal cholesterol metabolism in the Smith-Lemli-Opitz syndrome: report of clinical and biochemical findings in four patients and treatment in one patient. Am J Med Genet 50: 347-352.

Jezela-Stanek A, Ciara E, Malunowicz EM, et al (2008) Mild Smith-Lemli-Opitz syndrome: further delineation of 5 Polish cases and review of the literature. Eur J Med Genet 51: 124-140.

Jira P (2013) Cholesterol metabolism deficiency. Handbook of clinical neurology 113: 1845-1850.

Jong Hee C, Ki Joong K, Yong Seung H, Ki CS, Kim JW (2007) Identification of a novel DHCR7 mutation in a Korean patient with Smith-Lemli-Opitz syndrome. J Child Neurol 22: 1297-1300.

Kabra M, Gulati S, Kaur M, et al (2000) I-cell disease (Mucolipidosis II). Indian J Pediatr 67: 683-687.

Kelly MN, Tuli SY, Tuli SS, Stern MA, Giordano BP (2015) Brothers with Smith-Lemli-Opitz syndrome. J Pediatr Health Care 29: 97-103.

Kirkpatrick K, Ellwood J, Walker RW (2012) Mucopolysaccharidosis type I (Hurler syndrome) and anesthesia: the impact of bone marrow transplantation, enzyme replacement therapy, and fiberoptic intubation on airway management. Paediatr Anaesth 22: 745-751.

Kloska A, Bohdanowicz J, Konopa G, et al (2005) Changes in hair morphology of mucopolysaccharidosis I patients treated with recombinant human alpha-L-iduronidase (laronidase, Aldurazyme). Am J Med Genet A 139: 199-203.

Kosho T, Okamoto N, Ohashi H, et al (2013) Clinical correlations of mutations affecting six components of the SWI/SNF complex: detailed description of 21 patients and a review of the literature. Am J Med Genet A 161A: 1221-1237.

Krakowiak PA, Nwokoro NA, Wassif CA, et al (2000) Mutation analysis and description of sixteen RSH/Smith-Lemli-Opitz syndrome patients: polymerase chain reaction-based assays to simplify genotyping. Am J Med Genet 94: 214-227.

Kretzer FL, Hittner HM, Mehta RS (1981) Ocular manifestations of the Smith-Lemli-Opitz syndrome. Arch Ophthalmol 99: 2000-2006.

Kula RW, Shafiq SA, Sher JH, Qazi QH (1984) I-cell disease (mucolipidosis II). Differential expression in satellite cells and mature muscle fibers. J Neurol Sci 63: 75-84.

Kumar S, Suthar R, Panigrahi I (2012) Hypercortisolism and hypothyroidism in an infant with Smith-Lemli-Opitz syndrome. J Pediatr Endocrinol Metab 25: 1001-1005.

Kurihara M, Kumagai K, Goto K, Imai M, Yagishita S (1992) Severe type Hunter's syndrome. Polysomnographic and neuropathological study. Neuropediatrics 23: 248-256.

Langius FA, Waterham HR, Romeijn GJ, et al (2003) Identification of three patients with a very mild form of Smith-Lemli-Opitz syndrome. Am J Med Genet A 122A: 24-29.

Laraway S, Breen C, Mercer J, Jones S, Wraith JE (2013) Does early use of enzyme replacement therapy alter the natural history of mucopolysaccharidosis I? Experience in three siblings. Mol Genet Metab 109: 315-316.

Leroux S, Muller JB, Boutaric E, et al (2014) [Hurler syndrome: early diagnosis and treatment]. Arch Pediatr 21: 501-506.

Leroy JG, Sillence D, Wood T, et al (2014) A novel intermediate mucolipidosis II/IIIalphabeta caused by GNPTAB mutation in the cytosolic N-terminal domain. Eur J Hum Genet 22: 594-601.

Loffler J, Trojovsky A, Casati B, Kroisel PM, Utermann G (2000) Homozygosity for the W151X stop mutation in the delta7-sterol reductase gene (DHCR7) causing a lethal form of Smith-Lemli-Opitz syndrome: retrospective molecular diagnosis. Am J Med Genet 95: 174-177.

Lonardo F, Di Natale P, Lualdi S, et al (2014) Mucopolysaccharidosis type II in a female patient with a reciprocal X;9 translocation and skewed X chromosome inactivation. Am J Med Genet A 164A: 2627-2632.

Lorinez AE, Montes LF (1975) Mucopolysaccharidosis (type I Hurler-Scheie compound). J Cutan Pathol 2: 214-215.

Lowry RB, Yong SL (1980) Borderline normal intelligence in the Smith-Lemli-Opitz (RSH) syndrome. Am J Med Genet 5: 137-143.

Luderschmidt C, Schill WB, Burg D, von Figura K, Hubner G, Pongratz D (1979) [Mucopolysaccharidosis I-S (Scheie's disease) (author's transl)]. Dtsch Med Wochenschr 104: 1482-1487.

Mari F, Marozza A, Mencarelli MA, et al (2015) Coffin-Siris and Nicolaides-Baraitser syndromes are a common well recognizable cause of intellectual disability. Brain Dev 37: 527-536.

Martin R, Beck M, Eng C, et al (2008) Recognition and diagnosis of mucopolysaccharidosis II (Hunter syndrome). Pediatrics 121: e377-386.

Meinecke P, Blunck W, Rodewald A (1987) Smith-Lemli-Opitz syndrome. Am J Med Genet 28: 735-739.

Mendez HM, Pinto LI, Paskulin GA, Ricachnevsky N (1993) Is there a relationship between inborn errors of metabolism and extensive mongolian spots? Am J Med Genet 47: 456-457.

Miyake N, Abdel-Salam G, Yamagata T, et al (2016) Clinical features of SMARCA2 duplication overlap with Coffin-Siris syndrome. Am J Med Genet A 170: 2662-2670.

Morin G, Villemain L, Baumann C, Mathieu M, Blanc N, Verloes A (2003) Nicolaides-Baraitser syndrome: confirmatory report of a syndrome with sparse hair, mental retardation, and short stature and metacarpals. Clin Dysmorphol 12: 237-240.

Mossman J, Blunt S, Stephens R, Jones EE, Pembrey M (1983) Hunter's disease in a girl: association with X:5 chromosomal translocation disrupting the Hunter gene. Arch Dis Child 58: 911-915.

Mueller P, Moeckel A, Daehnert I (2006) Severe dilated cardiomyopathy as an unusual finding in a young infant with mucolipidosis type 2. Images Paediatr Cardiol 8: 1-6.

Mutesa L, Muganga N, Lissens W, et al (2007) Molecular analysis in two siblings African patients with severe form of Hunter Syndrome: identification of a novel (p.Y54X) nonsense mutation. J Trop Pediatr 53: 434-437.

Muzzin KB, Harper LF (2003) Smith-Lemli-Opitz syndrome: a review, case report and dental implications. Spec Care Dentist 23: 22-27.

Nayak DR, Balakrishnan R, Adolph S (1998) Endoscopic adenoidectomy in a case of Scheie syndrome (MPS I S). Int J Pediatr Otorhinolaryngol 44: 177-181.

Nelson J, Carson D (1989) Pituitary function studies in a case of mild Hunter's syndrome (MPS IIB). J Med Genet 26: 731-732.

Nezarati MM, Loeffler J, Yoon G, et al (2002) Novel mutation in the Delta-sterol reductase gene in three Lebanese sibs with Smith-Lemli-Opitz (RSH) syndrome. Am J Med Genet 110: 103-108.

Nicolaides P, Baraitser M (1993) An unusual syndrome with mental retardation and sparse hair. Clin Dysmorphol 2: 232-236.

Nowaczyk MJ, Irons MB (2012) Smith-Lemli-Opitz syndrome: phenotype, natural history, and epidemiology. Am J Med Genet C Semin Med Genet 160C: 250-262.

Nowaczyk MJ, Tan M, Hamid JS, Allanson JE (2012) Smith-Lemli-Opitz syndrome: Objective assessment of facial phenotype. Am J Med Genet A 158A: 1020-1028.

Nwokoro NA, Mulvihill JJ (1997) Cholesterol and bile acid replacement therapy in children and adults with Smith-Lemli-Opitz (SLO/RSH) syndrome. Am J Med Genet 68: 315-321.

Oghan F, Harputluoglu U, Guclu E, Guvey A, Turan N, Ozturk O (2007) Permanent t-tube insertion in two patients with Hurler's syndrome. Int J Audiol 46: 94-96.

Opitz JM, Furtado LV (2012) The RSH/"Smith-Lemli-Opitz" syndrome: historical footnote. Am J Med Genet C Semin Med Genet 160C: 242-249.

Orii T, Sukegawa K, Minami R, Matsuura Y, Tsugawa S (1976) Atypical Hurler syndrome without alpha-L-iduronidase deficiency. Tohoku J Exp Med 120: 113-123.

Pankau R, Partsch CJ, Funda J, Sippell WG (1992) Hypothalamic-pituitary-gonadal function in two infants with Smith-Lemli-Opitz syndrome. Am J Med Genet 43: 513-516.

Pasqualim G, Ribeiro MG, da Fonseca GG, et al (2015) p.L18P: a novel IDUA mutation that causes a distinct attenuated phenotype in mucopolysaccharidosis type I patients. Clin Genet 88: 376-380.

Pina-Aguilar RE, Zaragoza-Arevalo GR, Rau I, et al (2013) Mucopolysaccharidosis type II in a female carrying a heterozygous stop mutation of the iduronate-2-sulfatase gene and showing a skewed X chromosome inactivation. Eur J Med Genet 56: 159-162.

Porter FD (2006) Cholesterol precursors and facial clefting. J Clin Invest 116: 2322-2325.

Porter FD (2008) Smith-Lemli-Opitz syndrome: pathogenesis, diagnosis and management. Eur J Hum Genet 16: 535-541.

Porter FD, Herman GE (2011) Malformation syndromes caused by disorders of cholesterol synthesis. J Lipid Res 52: 6-34.

Pourjavan S, Fryns JP, Van Hove JL, Poorthuis BJ, Casteels I (2002) Ophthalmological findings in a patient with mucolipidosis III (pseudo-hurler polydystrophy). A case report. Bull Soc Belge Ophtalmol: 19-24.

Prystowsky SD, Maumenee IH, Freeman RG, Herndon JH, Jr., Harrod MJ (1977) A cutaneous marker in the Hunter syndrome a report of four cases. Arch Dermatol 113: 602-605.

Puiu M, Chirita-Emandi A, Dumitriu S, Arghirescu S (2013) Hunter syndrome follow-up after 1 year of enzyme-replacement therapy. BMJ Case Rep 2013.

Rayamajhi A, Pokharel PJ, Chapagain R, Rayamajhi AK (2013) Mucopolysaccharidosis type II with inguinal hernia. J Nepal Health Res Counc 11: 293-295.

Roubicek M, Gehler J, Spranger J (1985) The clinical spectrum of alpha-L-iduronidase deficiency. Am J Med Genet 20: 471-481.

Ruvalcaba RH, Reichert A, Smith DW (1968) Smith-Lemli-Opitz syndrome. Case report. Arch Dis Child 43: 620-623.

Ryan AK, Bartlett K, Clayton P, et al (1998) Smith-Lemli-Opitz syndrome: a variable clinical and biochemical phenotype. J Med Genet 35: 558-565.

Sanjurjo-Crespo P (2007) [Clinical aspects of mucopolysaccharidosis type II]. Rev Neurol 44 Suppl 1: S3-6.

Santen GW, Aten E, Vulto-van Silfhout AT, et al (2013) Coffin-Siris syndrome and the BAF complex: genotype-phenotype study in 63 patients. Hum Mutat 34: 1519-1528.

Sati A, Ramappa M, Chaurasia S, Prasad SM (2014) Deep anterior lamellar keratoplasty in case of Hurler-Scheie syndrome. BMJ Case Rep 2014.

Smith DW, Lemli L, Opitz JM (1964) A Newly Recognized Syndrome of Multiple Congenital Anomalies. J Pediatr 64: 210-217.

Sood S, Giacoia GP, Tunnessen WW, Jr. (1994) Picture of the month. Smith-Lemli-Opitz syndrome. Arch Pediatr Adolesc Med 148: 1189-1190.

Sousa SB, Abdul-Rahman OA, Bottani A, et al (2009) Nicolaides-Baraitser syndrome: Delineation of the phenotype. Am J Med Genet A 149A: 1628-1640.

Sousa SB, Hennekam RC, Nicolaides-Baraitser Syndrome International C (2014) Phenotype and genotype in Nicolaides-Baraitser syndrome. Am J Med Genet C Semin Med Genet 166C: 302-314.

Spellacy E, Bankes JL, Crow J, Dourmashkin R, Shah D, Watts RW (1980) Glaucoma in a case of Hurler disease. Br J Ophthalmol 64: 773-778.

Spranger J, Cantz M, Gehler J, Liebaers I, Theiss W (1978) Mucopolysaccharidosis II (Hunter disease) with corneal opacities. Report on two patients at the extremes of a wide clinical spectrum. Eur J Pediatr 129: 11-16.

Sprigz RA, Doughty RA, Spackman TJ, et al (1978) Neonatal presentation of I-cell disease. J Pediatr 93: 954-958.

Starck L, Bjorkhem I, Ritzen EM, Nilsson BY, von Dobeln U (1999) Beneficial effects of dietary supplementation in a disorder with defective synthesis of cholesterol. A case report of a girl with Smith-Lemli-Opitz syndrome, polyneuropathy and precocious puberty. Acta Paediatr 88: 729-733.

Stevenson RE, Howell RR, McKusick VA, et al (1976) The iduronidase-deficient mucopolysaccharidoses: clinical and roentgenorgraphic features. Pediatrics 57: 111-122.

Tajima G, Sakura N, Kosuga M, Okuyama T, Kobayashi M (2013) Effects of idursulfase enzyme replacement therapy for Mucopolysaccharidosis type II when started in early infancy: comparison in two siblings. Mol Genet Metab 108: 172-177.

Tang S, Hughes E, Lascelles K, Euro ERESmaewg, Simpson MA, Pal DK (2017) New SMARCA2 mutation in a patient with Nicolaides-Baraitser syndrome and myoclonic astatic epilepsy. Am J Med Genet A 173: 195-199.

Tatapudi R, Gunashekhar M, Raju PS (2011) Mucopolysaccharidosis type I Hurler-Scheie syndrome: A rare case report. Contemp Clin Dent 2: 66-68.

Thomas GH, Miller CS, Toomey KE, et al (1982) Two clonal cell populations (mosaicism) in a 46,XY male with mucolipidosis II (I-cell disease)--an autosomal recessive disorder. Am J Hum Genet 34: 611-622.

Tiede S, Cantz M, Spranger J, Braulke T (2006) Missense mutation in the N-acetylglucosamine-1-phosphotransferase gene (GNPTA) in a patient with mucolipidosis II induces changes in the size and cellular distribution of GNPTG. Hum Mutat 27: 830-831.

Tiede S, Muschol N, Reutter G, Cantz M, Ullrich K, Braulke T (2005) Missense mutations in N-acetylglucosamine-1-phosphotransferase alpha/beta subunit gene in a patient with mucolipidosis III and a mild clinical phenotype. Am J Med Genet A 137A: 235-240.

Tsurusaki Y, Okamoto N, Ohashi H, et al (2012) Mutations affecting components of the SWI/SNF complex cause Coffin-Siris syndrome. Nat Genet 44: 376-378.

Tucci A, Ronzoni L, Arduino C, Salmin P, Esposito S, Milani D (2016) The p.Phe174Ser mutation is associated with mild forms of Smith Lemli Opitz Syndrome. BMC Med Genet 17: 22.

Tylki-Szymanska A, Czartoryska B, Groener JE, Lugowska A (2002) Clinical variability in mucolipidosis III (pseudo-Hurler polydystrophy). Am J Med Genet 108: 214-218.

Tylki-Szymanska A, Jurecka A, Zuber Z, Rozdzynska A, Marucha J, Czartoryska B (2012) Enzyme replacement therapy for mucopolysaccharidosis II from 3 months of age: a 3-year follow-up. Acta Paediatr 101: e42-47.

Tzouvelekis G, Antoniades K, Batma A, Nanas C (1991) Smith-Lemli-Opitz syndrome in female, monozygotic twins. Clin Genet 40: 229-232.

Van Houdt JK, Nowakowska BA, Sousa SB, et al (2012) Heterozygous missense mutations in SMARCA2 cause Nicolaides-Baraitser syndrome. Nat Genet 44: 445-449, S441.

Vas L, Naregal F (2000) Failed epidural anaesthesia in a patient with Hurler's disease. Paediatr Anaesth 10: 95-98.

Ward C, Singh R, Slade C, et al (1993) A mild form of mucolipidosis type III in four Baluch siblings. Clin Genet 44: 313-319.

Winters PR, Harrod MJ, Molenich-Heetred SA, Kirkpatrick J, Rosenberg RN (1976) alpha-L-iduronidase deficiency and possible Hurler-Scheie genetic compound. Clinical, pathologic, and biochemical findings. Neurology 26: 1003-1007.

Wolff D, Endele S, Azzarello-Burri S, et al (2012) In-Frame Deletion and Missense Mutations of the C-Terminal Helicase Domain of SMARCA2 in Three Patients with Nicolaides-Baraitser Syndrome. Mol Syndromol 2: 237-244.

Wraith JE, Cooper A, Thornley M, et al (1991) The clinical phenotype of two patients with a complete deletion of the iduronate-2-sulphatase gene (mucopolysaccharidosis II--Hunter syndrome). Hum Genet 87: 205-206.

Wraith JE, Scarpa M, Beck M, et al (2008) Mucopolysaccharidosis type II (Hunter syndrome): a clinical review and recommendations for treatment in the era of enzyme replacement therapy. Eur J Pediatr 167: 267-277.

Yamada H, Ohya M, Higeta T, Kinoshita S (1987) Craniosynostosis and hydrocephalus in I-cell disease (mucolipidosis II). Childs Nerv Syst 3: 55-57.

Yatziv S, Epstein CJ (1977) Hunter syndrome presenting as macrocephaly and hydrocephalus. J Med Genet 14: 445-447.

Yoskovitch A, Tewfik TL, Brouillette RT, Schloss MD, Der Kaloustian VM (1998) Acute airway obstruction in Hunter syndrome. Int J Pediatr Otorhinolaryngol 44: 273-278.

Young ID, Harper PS (1982) Mild form of Hunter's syndrome: clinical delineation based on 31 cases. Arch Dis Child 57: 828-836.

Yuksel A, Kayserili H, Gungor F (2007) Short femurs detected at 25 and 31 weeks of gestation diagnosed as Leroy I-cell disease in the postnatal period: a report of two cases. Fetal Diagn Ther 22: 198-202.

Zabel RW, MacDonald IM, Mintsioulis G, Addison DJ (1989) Scheie's syndrome. An ultrastructural analysis of the cornea. Ophthalmology 96: 1631-1638.

Zolkipli Z, Noimark L, Cleary MA, Owens C, Vellodi A (2005) Temporomandibular joint destruction in mucolipidosis type III necessitating gastrostomy insertion. Eur J Pediatr 164: 772-774.
