# Supplementary figures and images for: Advances in computer-assisted syndrome recognition by the example of inborn errors of metabolism
Source: J Inherit Metab Dis. 2018 Apr 5;41(3):533–9. doi: 10.1007/s10545-018-0174-3 (PMC5959962; doi:10.1007/s10545-018-0174-3)

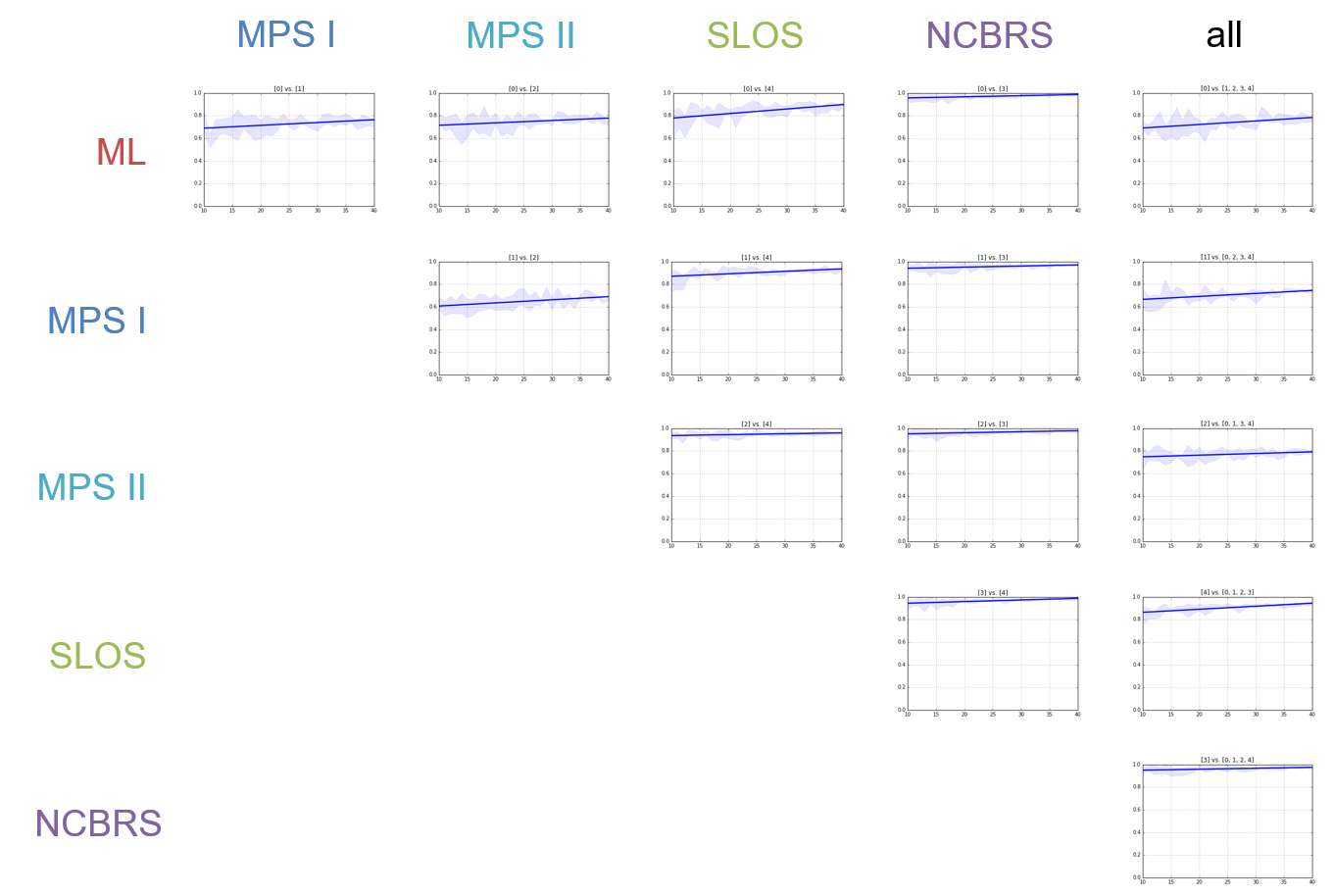

Supplement: Supplementary file 2 — (PNG 170 kb) [file 10545_2018_174_MOESM2_ESM.png]
